# Fast Field-Cycling Nuclear Magnetic Resonance Relaxometry of Perfluorosulfonic Acid Ionomers and Their Perfluorosulfonyl Fluoride Precursors Membranes

**DOI:** 10.3390/molecules29112552

**Published:** 2024-05-29

**Authors:** Makoto Yamaguchi, Seiichi Kuroda, Takahiko Asaoka, Kazuhiko Shinohara

**Affiliations:** FC-Cubic (Fuel Cell Cutting-Edge Research Center), Technology Research Association, 3147 Shimomukoyama-cho, Kofu 400-1507, Yamanashi, Japan

**Keywords:** spin-lattice relaxation, microphase separation, defect diffusion model, reptation

## Abstract

The spin-lattice relaxation rates (*R*_1_) of fluorine nuclei in perfluorosulfonic acid (PFSA) ionomer membranes and their precursor solid perfluorosulfonyl fluoride (PFSF) were measured by fast field-cycling (FFC) NMR relaxometry. The XRD profiles of PFSA and PFSF are similar and show a characteristic peak, indicating the alignment of main chains. While the SAXS profiles of the PFSA membranes show two peaks, those of the solid PFSF lack the ionomer peak which is characteristic of hydrophilic side chains in the PFSA ionomer membranes. The Larmor frequency dependence of *R*_1_ obeys power law and the indices are dependent on the sample and temperature. The indices of the PFSA membranes change from −1/2 to −1 along with the Larmor frequency and temperature dependence decrease, which is consistent with the generalized defect diffusion model. Estimated activation energies are in good agreement with those obtained from dynamical mechanical analysis and dielectric spectroscopy, indicating the segmental motion of the backbones as the common origin of these observations. On the other hand, the index changes to −3/4 in the case of the PFSFs, which has been predicted by the reptation model.

## 1. Introduction

Perfluorosulfonic acid (PFSA) ionomers such as Nafion have been widely used in polymer electrolyte fuel cells (PEFCs) as proton exchange membranes and ionomers in electrodes [1]. They are copolymers of tetrafluoroethylene and perfluoro-oxy-alkenyl sulfonic acid and their polytetrafluoroethylene (PTFE)-like hydrophobic backbones and hydrophilic sulfonic acid side chain terminal induce microphase separation, as was suggested from the ionomer peaks at around *q* = 2 nm^−1^ in the small-angle X-ray scattering (SAXS) profiles. It has been recognized that this microphase separation is one of the keys for the high proton conductivity of PFSA ionomer membranes as the connectivity of the hydrophilic channels is better at the same water content than other types of proton-conducting membranes. The self-diffusion coefficients of water have also been measured using pulsed-field gradient nuclear magnetic resonance (PFG-NMR) techniques. While the diffusion coefficient increases with water content, which is in parallel with the proton conductivity, its activation energy is almost constant at high water content and close to that of bulk water. As the water content decreases, the hydrophilic regions become disconnected and the diffusivity drops. This accompanies increased activation energy as proton migration becomes coupled with the dynamics of the polymer chains [2].

NMR has also been applied to investigate the dynamics of the polymers in PFSA membranes: Boyle et al. measured the temperature dependence of ^19^F *T*_1_ at 40 MHz and correlated the results with the relaxations from dynamic mechanical analysis (DMA) measurements [3]. Page et al. measured sold-state ^19^F NMR spectra with relatively low spinning rates and measured the temperature dependence of *T*_1_ and *T*_1ρ_ [4,5]. Chen and Schmidt-Rohr measured two-dimensional ^19^F-^13^C solid-state NMR spectra with high sample-spinning rates and derived peak widths of the deconvoluted peaks from different sites, which are related to their degree of disorder and mobility [6]. In their following paper, they analyzed ^19^F-^13^C dipolar coupling and chemical shift anisotropies and deduced the movement of the backbone axis which is enhanced by hydration [7].

Among various NMR methodologies, fast field-cycling (FFC) is one of the most efficient methods for NMR relaxometry [8]. Spin-lattice relaxation rates are measured by changing the static magnetic field and thus the obtained relaxation rate profiles are correlated with spectral density functions. In fact, the method has already been applied to PFSA membranes to probe the dynamics of protons [9,10,11]. In contrast, as far as we know, the method has not been applied for the relaxation of ^19^F nuclei in the PFSA ionomer membranes. Based on the results of the FFC-NMR on polymeric materials, it is worthwhile to apply the method to probe the dynamics of PFSA ionomers.

In this study, we also employed perfluoroalkyl sulfonyl fluoride (PFSF) membranes as reference materials of the PFSA membranes. PFSF is a precursor of PFSA and its side chains are terminated with sulfonyl fluoride (SO_2_F). In contrast to PFSA membranes, PFSF membranes are able to be melt-processed. They do not absorb water and show no microphase separation. These differences between PFSA and PFSF are reflected in their dynamics, as shown by the significant lowering of the α-transition temperature from the PFSA to the corresponding PFSF [4,12,13]. It is expected that ^19^F FFC-NMR would help in further understanding the polymer dynamics of these materials.

## 2. Materials and Methods

**Scheme 1:**
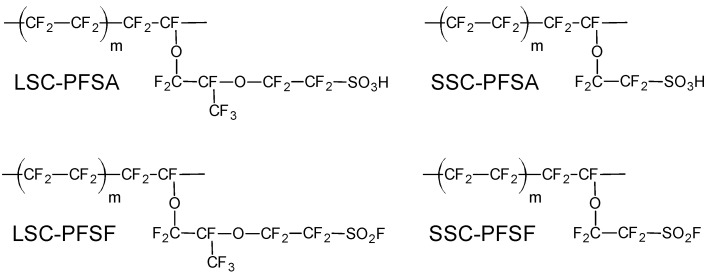
Chemical structure of the studied materials.

LSC-PFSA Nafion NR212 membrane (EW = 1100 g mol^−1^; thickness = 0.05 mm) was purchased from Chemours (Wilmington, NC, USA). SSC-PFSA Aquivion E98-05S (EW = 980 g mol^−1^; thickness = 0.05 mm) and E79-12S (EW = 790 g mol^−1^; thickness = 0.12 mm) membranes were purchased from Solvay (Brussels, Belgium). LSC-PFSF Nafion R-1100 membrane (EW = 1100 g mol^−1^; thickness = 0.05 mm) was purchased from Alfa Aesar (Ward Hill, MA, USA). SSC-PFSF Aquivion P98-SO_2_F pellet (EW = 980 g mol^−1^; 2 mm cylinder) was purchased from Sigma-Aldrich (Saint Louis, MO, USA). Their chemical structures are shown in Scheme 1. Perfluorotetrafluoroethylene (PTFE) was cut from a seal tape roll (thickness = 0.1 mm). 1-propanol (HPLC grade, 99.7%) was purchased from Wako Pure Chemical Industries, Ltd. (Osaka, Japan).

The X-ray diffraction (XRD) profiles were measured using an Ultima IV X-ray diffractometer (Rigaku, Tokyo, Japan) with a Cu Kα (λ = 1.54 Å) radiation source operated at 40 kV and 40 mA. Membrane samples were directly placed on a sample holder except P98-SO_2_F, which was pulverized and placed on a slide glass. The α_2_ contribution was removed from the measured data by the Rachinger correction [14].

Small-angle X-ray scattering (SAXS) profiles of membranes were measured using a NanoViewer (Rigaku, Tokyo, Japan) with a Cu Kα radiation source operated at 40 kV and 30 mA. A P98-SO_2_F membrane sample (thickness ~ 0.11 mm) was fabricated by hot pressing the pellets (DJK Corporation, Yokohama, Japan). A humidification chamber and a controller were used for measurement under controlled relative humidity at room temperature. The camera length was set to 660 mm. SAXS images were obtained using Pilatus 100K (Dectris, Baden, Switzerland) in transmission mode by accumulating signals for 60 min. Most of the SAXS images were isotropic and they were azimuthally integrated to obtain one-dimensional profiles. The R-1100 membrane is exceptional as its SAXS images were highly anisotropic, as shown in Figure S1 in the Supplementary Materials. One-dimensional profiles were obtained along the direction where the scattering is the strongest. Model function fitting of the one-dimensional profiles was performed using a SasView program package [15].

**Figure 1:**
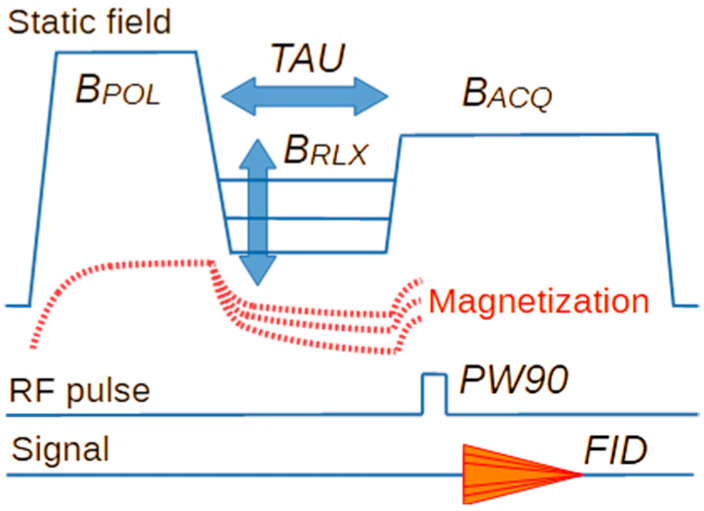
Pulse sequence for the spin-lattice relaxation rate measurement with magnetization pre-polarization.

The spin-lattice relaxation rates (*R*_1_ = 1/*T*_1_) of ^19^F nuclei were measured using a fast field-cycling NMR relaxometer (Spinmaster FFC-2000, Stelar s.r.l., Mede, Italy). About 0.25–1 g of each sample was placed in a glass tube (10 mm o.d.) and the tube was capped without evacuation. The sample temperature was controlled with nitrogen gas flow from 300 to 393 K. A typical pulse sequence with magnetization pre-polarization is schematically illustrated in Figure 1. In the beginning, the static magnetic field *B*_pol_ = 0.587 T generated by the electromagnet of the system is applied to polarize the magnetization of the sample. Then, the magnetic field is quickly switched to *B*_rlx_ and the magnetization decays by spin-lattice relaxation at a lower magnetic field than *B*_pol_. After a certain period (tau) of *B*_rlx_, the static magnetic field is switched to *B*_acq_ = 0.407 T and the π/2 RF pulse is applied immediately to detect remaining magnetization as the FID (Free Induction Decay) signal. The measurement was repeated with the same *B*_rlx_ and tau and the accumulation number varied from 4 to 256. The tau was changed in 16 steps at each *B*_rlx_ and the spin-lattice relaxation rate constant *R*_1_ was calculated by assuming single exponential decay of the FID signal intensities. This process was repeated by changing *B*_rlx_ from 0.0117 mT to 0.939 T at the highest, which correspond to a ^1^H Larmor frequency of about 5 kHz to 40 MHz. The *B*_pol_ was not applied when *B*_rlx_ > 0.234 T.

## 3. Results

### 3.1. XRD

**Figure 2:**
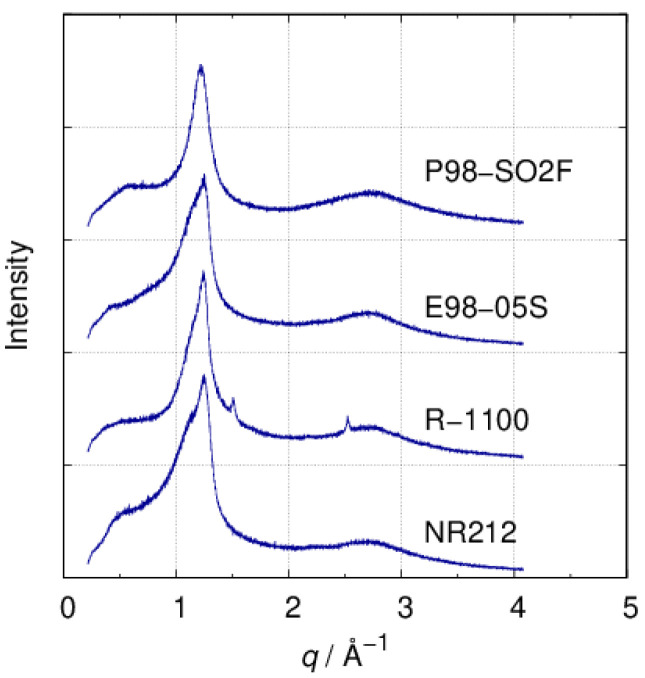
X-ray diffraction profiles of Nafion (PFSA: NR212, PFSF: R-1100) and Aqiivion (PFSA: P98-05S, PFSF: P98-SO_2_F).

Figure 2 shows the XRD profiles of the PFSF and the PFSA samples. All show the strongest peak at around *q* = 1.2 Å^−1^. They are slightly broader in the PFSA than the PFSF and asymmetric with a shoulder on the low-q side, which is usually assigned as crystalline and amorphous regions. We did not quantify the intensity ratio of the two peaks as it was difficult to perform the baseline correction. A broad peak at around *q* = 2.8 Å^−1^ is also observed in all the samples. These two peaks are commonly observed in the XRD profiles in previous papers on the PFSA membranes [1]. Starkweather assigned the peaks to (100) and (101) reflections from the hexagonal unit cell (*a* = 5.8 and *c* = 2.6 Å) [16]. On the other hand, van der Heijden et al. assigned these peaks to the (200) and (211) planes of the orthorhombic unit cell (*a* = 9.9; *b* = 5.6; and *c* = 2.8 Å) [17]. They derived the cell parameters by fitting the peak positions of Nafion-SO_2_F, which shows additional sharp peaks. They claimed their orthorhombic unit cell model can explain the appearance of peaks between 1.5 and 2 Å^−1^, while the hexagonal unit cell model by Starkweather fails. However, the orthorhombic unit cell model can be derived by slight distortion from the hexagonal unit cell model and both models explain the peak at around 1.2 Å^−1^ as the backbones aligned along the *c*-axis.

### 3.2. SAXS

**Figure 3:**
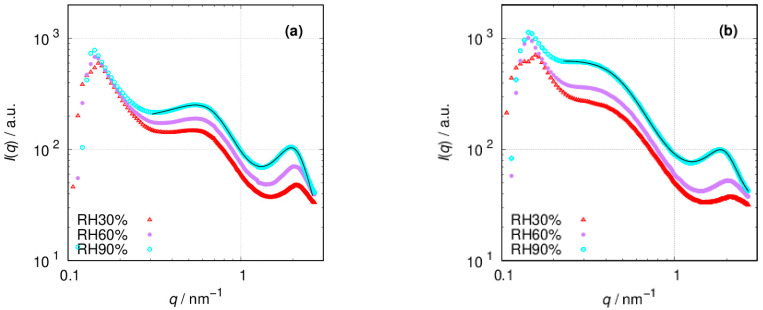
SAXS profiles of PFSA membranes under humidification: (**a**) NR212 and (**b**) E98-05S.

Figure 3 shows the SAXS profiles of the LSC and SSC PFSA membranes under controlled relative humidity. Two peaks appear at around 2 and 0.6 nm^−1^ in the SAXS profiles of the NR212 membrane in the left panel, while the position of the latter peak is slightly shifted to 0.5 nm^−1^ in the case of E98-05S in the right panel. The former is the so-called “ionomer peak” characteristic of the PFSA ionomer membranes due to the certain ordered structure of the hydrophilic domains. The latter is called the “matrix knee”, which is supposed to originate from the ordered domains of the polymer backbones [17]. As the water uptake increases with relative humidity, the ionomer peak becomes stronger and shifts to lower *q*. On the other hand, the matrix knee is little affected with relative humidity.

**Figure 4:**
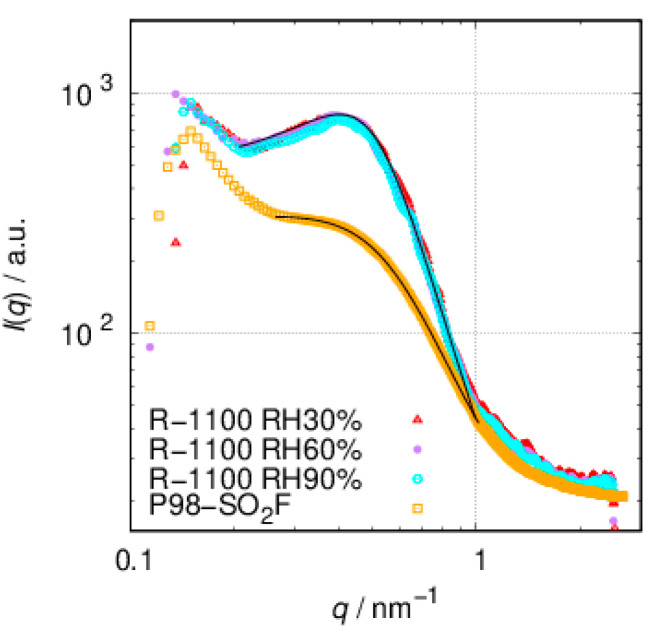
SAXS profiles of PFSF membranes under humidification.

In contrast, the ionomer peak is absent in the SAXS profiles of the LSC and SSC PFSF membranes in Figure 4. This is expected from the absence of the hydrophilic acid sites in the side chains and the SAXS profiles of the LSC R-1100 membrane show no or little change with relative humidity. A peak at 0.4 nm^−1^ is much stronger than the “matrix knee” of the NR212 membrane. Bundles of the aggregates of the backbones are more developed in the R-1100 membranes. This SAXS profile is very similar to that of the membranes with the side chains terminated with sulfonyl chloride (SO_2_Cl) [18]. The SAXS profile of the SSC P98-SO_2_F membrane also shows a peak at the same position but it is much weaker than the LSC R-1100.

The broad peaks in the SAXS profiles were fitted with the Teubner–Strey model [19], which has been successfully applied to fit the SANS profiles of the fully hydrated Nafion membrane [20]. The periodicity (*d*) and correlation length (*ξ*) derived from fittings are summarized in Table 1 and fitted curves are plotted with lines in Figure 3 and Figure 4. Two functions with different parameters are employed to fit two peaks in the SAXS profiles of the PFSA membranes. The values of the NR212 membrane (RH90%) are slightly smaller than those derived from SANS of the fully hydrated NR212 membranes.

### 3.3. FFC-NMR

#### 3.3.1. PFSA

**Figure 5:**
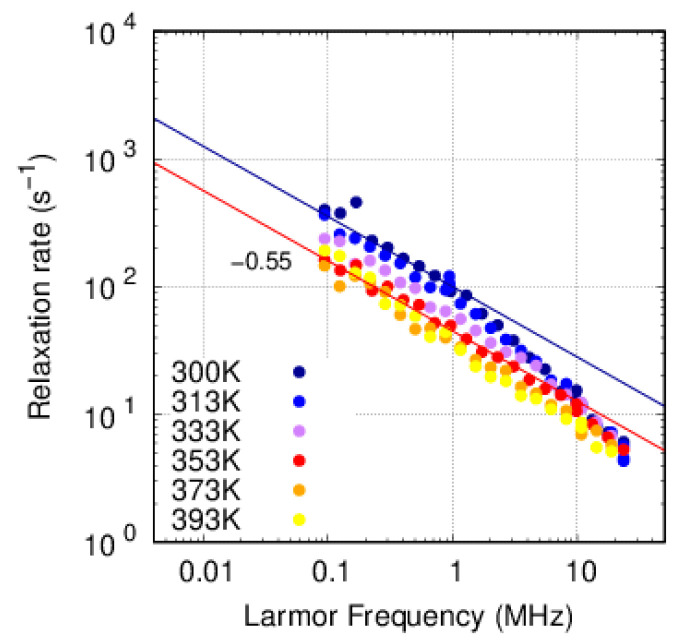
Spin-lattice relaxation rates of NR212 membrane.

Figure 5 shows spin-lattice relaxation rates (*R*_1_) of the Nafion membranes. This and the other graphs shown below on *R*_1_ are plotted against *B*_rlx_, which is represented as ^19^F Larmor frequency. The relaxation rates at certain temperatures decrease linearly with Larmor frequency in a log–log plot, indicating power law dependence. At higher temperature, the index is approximately −0.55 throughout the measured frequency range, as indicated by the straight red line. While the decay rate at a certain Larmor frequency increases as the temperature decreases, the temperature dependence is smaller at a higher Larmor frequency. At 300 K, the dark blue line with the index −0.55 approximates the measured points only up to around 1 MHz.

**Figure 6:**
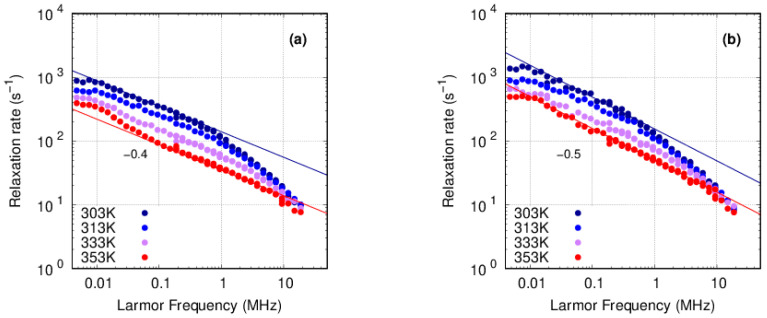
Spin-lattice relaxation rates of SSC PFSA membranes in air: (**a**) E98-05S and (**b**) E79-12S.

Figure 6 shows the spin-lattice relaxation rates of the SSC PFSA Aquivion membranes of two different EW values of 980 and 790 g mol^−1^. The membranes were left in ambient condition in the laboratory without any specific humidity control before measurement. Two straight lines obeying power law frequency dependence are plotted in these figures, with slightly different indices of −0.4 and −0.5 for E98-05S and D79-12S, respectively. While the measured data obey power law, the index changes from approximately −1/2 to −1 at around 1 MHz at 303 K. As the temperature increases, the relaxation rates decrease and the deflection point shifts to higher frequency. Temperature dependence is almost negligible above 10 MHz in E79-12S. Deviation from the power law dependence is also observed at the lower-frequency side in both samples.

The observed frequency dependence can be explained by the generalized defect diffusion model by Lenk as the spectral density function of Equation (1) [21].

(1) $$\ln J\left(\omega ,T\right)=\left(1-\frac{1}{2}\alpha \right)\ln\tau_{0}+\left(1-\frac{1}{2}\alpha \right)\frac{E_{a}}{kT}-\frac{1}{2}\alpha \ln\omega +const.$$

This equation was derived by assuming temperature dependence of Arrhenius type for the characteristic diffusion time *τ* = *τ*_0_ exp(−*E_a_*/*kT*). The power law frequency dependence arises from the third term and indices of −1/2 and −1 correspond to the case of α = 1 and 2, respectively. When α = 1, the second term becomes −*E_a_*/2*kT* and the spectral density function decreases with temperature, which are in accordance with the temperature dependence observed in Figure 5 and Figure 6 in the middle of the frequency range. When α = 2, the temperature dependence becomes absent and the power law index of frequency dependence changes to −1. These characteristics are observed at the highest-frequency side above 10 MHz in Figure 5 and Figure 6.

**Figure 7:**
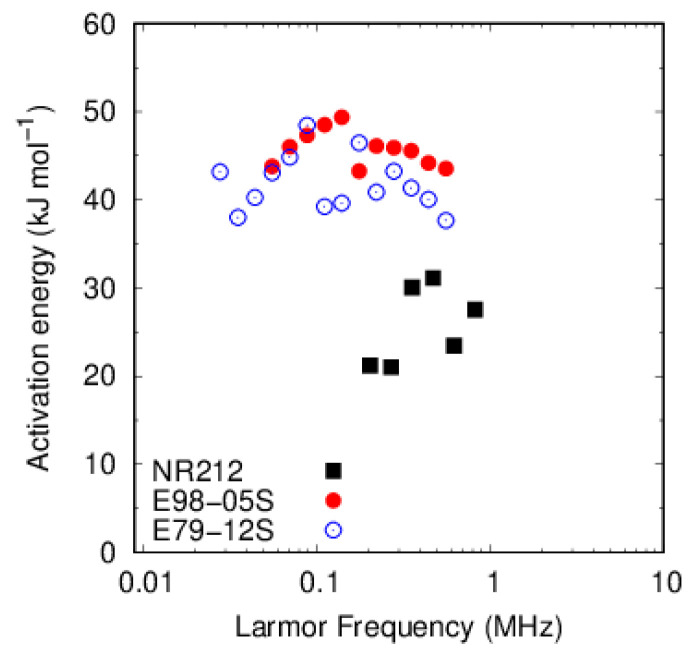
Apparent activation energies of spin-lattice relaxation rates of PFSA membranes.

Figure 7 shows the activation energies derived from Arrhenius plots of the measured data in the frequency range where α = 1 in Equation (1) holds. The frequency range was set from visual inspection of the plot where measured data are in parallel within the two straight lines with the index close to −0.5. Arrhenius plots of the relaxation rates (*R*_1_ vs. 1/*T*) are given in Figures S2 and S3 in the Supplementary Materials. While the activation energies of Nafion NR212 are 20–30 kJ mol^−1^, the activation energies of Aquivion membranes are 40–50 kJ mol^−1^. The average vales are 26, 46 and 42 kJ mol^−1^ for NR212, E98-05S and E79-12S, respectively. These activation energies fall within the range of the activation energies of around 40 kJ mol^−1^ obtained from DMA or dielectric spectroscopy as summarized by Kusoglu and Weber in their review [1].

**Figure 8:**
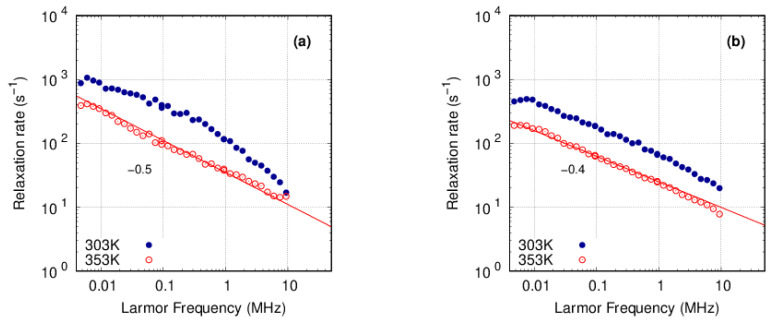
Spin-lattice relaxation rates of E79-12S membranes. (**a**) in water (**b**) in 1-propanol.

Figure 8 shows the spin-lattice relaxation rates of the E79-12S SSC PFSA membranes immersed in water or 1-propanol. In both cases, the relaxation rates are lower than those of the same membrane measured in air shown in Figure 6b. This implies the dipolar interaction between the polymer and solvent molecules does not give additional contribution to the spin-lattice relaxation process of fluorine atoms in the polymers. It is interesting to see that the relaxation profiles of the E79-12S in Figure 8a measured in water are almost identical to those of the E98-05S membranes in Figure 6a measured in air at the same temperature. The measured points obey power law as indicated by the straight line with the index of −0.5. On the other hand, the shape of the profiles does not change with temperature in the membrane immersed in 1-propanol. Both of the profiles at 303 and 353 K obey power law with the index of approximately −0.4 and the rates are slightly lower than those in water at the same temperature. According to Equation (1), this implies a smaller intrinsic defect diffusion time *τ*_0_ in the first term. It has been reported that Nafion dispersions become diffuse in water–alcohol mixtures [22,23] and their ^19^F-NMR spectra show narrower peaks in the dispersions with higher alcohol fractions [24]. Hydrophobic interaction between the perfluoroalkyl groups in the PFSA ionomer and the alkyl groups of aliphatic alcohols might have induced more randomized movements of the side chains and partially unfolded the partially aligned backbones.

#### 3.3.2. PFSF

**Figure 9:**
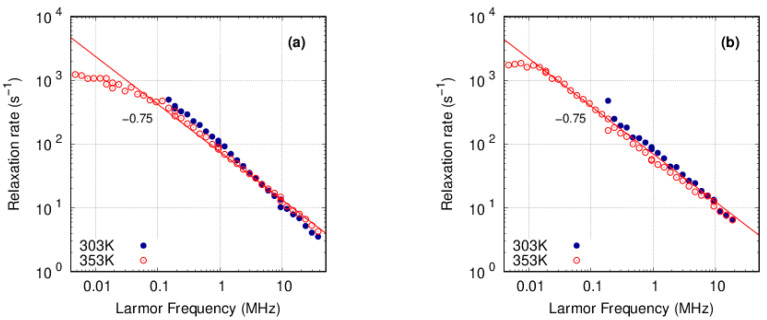
Spin-lattice relaxation rates of PFSF in air: (**a**) Nafion R-1100 and (**b**) P98-SO_2_F.

Figure 9 shows the spin-lattice relaxation rates of the LSC (Nafion R-1100) and SSC (P98-SO_2_F) PFSFs at 303 and 353 K. The relaxation rates obey power law at both temperatures and the indices change from −1 at 303 K to −3/4 at 353 K above 0.1 MHz with little changes in the relaxation rates. The slope becomes less steep below 0.1 MHz in the case of R-1100 and the index is close to −1/2. As the frequency becomes lower, the relaxation rates become less frequency-dependent and they are almost constant below 15 kHz. The power law with the index of −3/4 has been predicted by de Gennes [25] in the reptation model and the mean square displacement of polymers confined in a harmonic radial potential [26].

**Figure 10:**
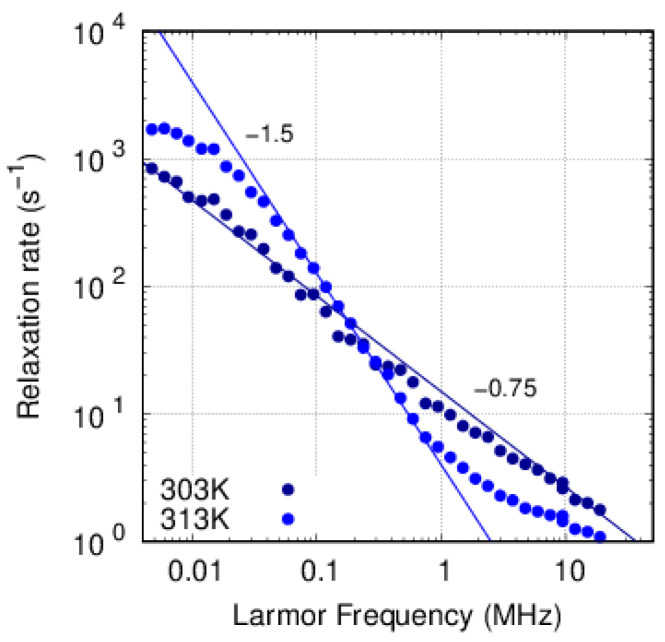
Spin-lattice relaxation rates of PTFE.

As the reptation model indicates that the relaxation is governed by the segmental motion of the polymer backbones, it is expected that PTFE having no side chains would show similar relaxation profiles. This supposition is confirmed by the spin-lattice relaxation profile of PTFE at 303 K in Figure 10. The measured data are fairly well approximated by the power law with the index −3/4 shown as the straight line, although certain deviation can be seen at around 1 MHz. Relaxation rates are lower than those of the PFSF membranes shown in Figure 8 at the same temperature. PTFE is in Form IV at this temperature and its screw axis symmetry is 15_7_ with segregated helix reversal defects [27]. It changes into Form I at 313 K with increased defect motion toward the planarity of CF_2_ groups. This change in the crystal form may be reflected in the relaxation profile, showing more pronounced frequency dependence with the index close to −1.5 from 0.05 to 0.5 MHz.

## 4. Discussion

As shown above, the frequency dependence of the spin-lattice relaxation rates of the solid PFSA and PFSF obeys power law. However, while the profiles of the PFSA membranes are explained by the defect diffusion model, those of the solid PFSF are consistent with the reptation model. As PFSF is the precursor of PFSA ionomers, the difference in the side chain terminals, which are transformed from sulfonyl fluoride in PFSF to sulfonic acid in PFSA, should be the origin of their frequency dependence. This change induces microphase separation as evidenced by the ionomer peak in the SAXS profiles of the PFSA membrane in Figure 3, which is absent in the PFSF in Figure 4. Solvent uptake in the hydrophilic region may facilitate the migration of polymer chains detached from the hydrophobic region. This change is reflected in the increased ratio of the amorphous to crystalline peaks in the XRD profiles of the PFSA compared to the PFSF shown in Figure 2. The portion of the polymer chain detached from a bundle of the backbones migrates along the chain as a defect to induce spin-lattice relaxation. This detachment of the main chains from the bundles is consistent with the results from solid-state NMR by Chen and Schmidt-Rohr [6]. The length of the detached portion may become longer when the sample is immersed in 1-propanol, which does shows constant slopes throughout the frequency range in Figure 8.

In contrast, hydrophilic regions are absent and the detachment of the polymer chains from the bundles is suppressed in the PFSF. As a certain periodicity is observed in the SAXS profiles, voids or low-density regions may be present in the PFSF where the movement of the polymer chains may be facilitated. However, the periodicity of the PFSF is much larger than the ionomer peaks of the PFSA and the movement perpendicular to the alignment of the backbones is restricted for the large part of the polymer chains. Under such circumstances, the movements of the polymer segments are only allowed along the direction of the alignment of the main chains, which is the same as the reptation of the polymer chains in a fictious tube in polymer melts. This reptation model predicts the relaxation profiles obeying power law with the index −3/4 as shown in Figure 9. The model also predicts that somewhat coherent motions of the polymer segments become dominant at lower frequencies and the index changes to −1/2 [28]. This change in the index was also observed in the case of the Nafion PFSF. It is interesting to note that “these laws have never been verified in experiments with bulk melts of entangled polymers” [28] (p. 487). The measurement temperature of 353 K is above the α-transition of both the LSC and SSC PFSFs [13]. Due to weak interaction between elongated perfluorinated helical chains aligned in parallel, one polymer chain could move within a tube formed by the neighboring chains.

As noted in the Introduction, the PFSA ionomers are used not only as the membranes but also in the catalyst layers as proton conductors. While this study focused on the membranes, the ^19^F FFC-NMR method can also be applied to the catalyst layer materials. In the catalyst layers, thin layers of ionomers cover carbon-supported platinum catalyst particles to mediate proton transport between the membrane and the catalyst particles. Due to the different geometry from the membrane and the confinement effect from the catalyst surface, the frequency dependence of the relaxation rates would become quite different from those reported here in the case of the PFSA membranes.

The thin layers of ionomers covering catalyst particles are also required to not inhibit oxygen diffusion to the catalyst surface. As catalyst layers are fabricated by the coating and subsequent drying of catalyst ink on a substrate, the size and shape of the ionomer particles and their changes during solvent evaporation have to be carefully controlled. The ^19^F FFC-NMR of the catalyst inks and ionomer dispersions at different concentrations would provide information on the structural changes in the ionomers during the catalyst layer fabrication process. The finite size of the dispersed particles would limit the range of the defect diffusion along the chain. Rotation of the particles also contributes to the spin-lattice relaxation and thus affects the frequency dependence of the relaxation rates.

Newly developed high-oxygen-permeability ionomers (HOPIs) for catalyst layers [29,30,31] would be the most interesting target for applications of the ^19^F FFC-NMR methods. These PFSA ionomers are synthesized with bulky cyclic monomers to prevent the formation of crystalline phases of the tetrafluoroethylene units in normal PFSA ionomers, since they act as barriers for oxygen diffusion. Certainly, the peaks from the crystalline phase are absent in their XRD and SAXS profiles. The nature of the diffusing defects and their motions along the polymer chains and thus the spin-lattice relaxation profiles should be quite different from those of the PFSA ionomers.

## 5. Conclusions

The spin-lattice relaxation rates of the PFSA membranes and their PFSF precursors were measured by fast field-cycling NMR relaxometry. While all the samples showed frequency dependence obeying power law, their indices and temperature dependences were different between PFSA and PFSF. The difference was attributed to the presence/absence of microphase separation in the PFSA/PFSF which induced different segmental motions of the polymer chains. It is expected that the temperature range of the FFC-NMR measurements will be extended in future works to correlate the results with the relaxation mechanisms by DMA or dielectric spectroscopy, which are usually probed with much wider temperature ranges.

## Figures and Tables

**Table 1:** Periodicity (*d*) and correlation length (*ξ*) in Teubner–Strey model.

	*d*/nm	*ξ*/nm
NR212 ^a^	9.9, 3.1	3.0, 1.8
E98-05S ^a^	16.2, 3.3	3.1, 2.0
R-1100 ^a^	13.8	4.7
P98-SO_2_F	14.4	2.7

^a^ RH90%.

## Data Availability

The data presented in this study are available on reasonable request from the corresponding author.

## Supplementary Materials

The following supporting information can be downloaded at: https://www.mdpi.com/article/10.3390/molecules29112552/s1: Figure S1: SAXS image of R-1100. Figures S2 and S3: Arrhenius plots of relaxation rates of the PFSA membranes.
