# Supplementary material for: Fast Field-Cycling Nuclear Magnetic Resonance Relaxometry of Perfluorosulfonic Acid Ionomers and Their Perfluorosulfonyl Fluoride Precursors Membranes
Source: Molecules. 2024 May 29;29(11):2552. doi: 10.3390/molecules29112552 (PMC11174070; doi:10.3390/molecules29112552)
Supplement: Supplementary file 1 [file molecules-29-02552-s001.zip › molecules-2950587-supplementary.pdf]

Article

Fast Field-Cycling Nuclear Magnetic Resonance Relaxometry of Perfluorosulfonic Acid Ionomers and Their Perfluorosulfonyl Fluoride Precursors Membranes

Makoto Yamaguchi *, Seiichi Kuroda, Takahiko Asaoka and Kazuhiko Shinohara

FC-Cubic (Fuel Cell Cutting-Edge Research Center), Technology Research Association, 3147 Shimomukoyama-cho, Kofu 400-1507, Yamanashi, Japan

* Correspondence: m-yamaguchi@fc-cubic.or.jp

Supplementary Material

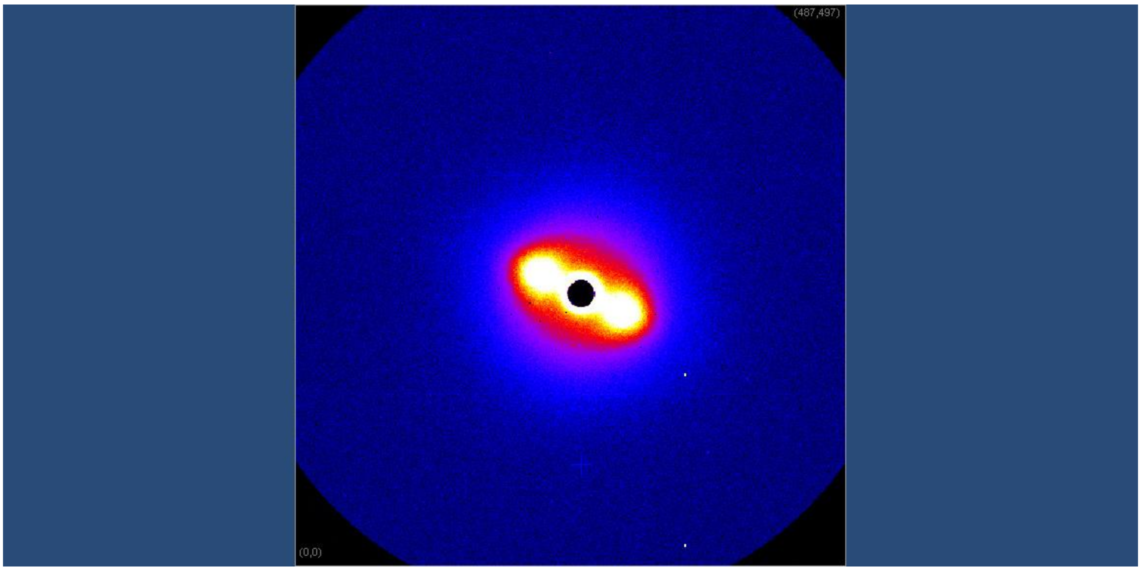

Figure S1. SAXS image of R-1100

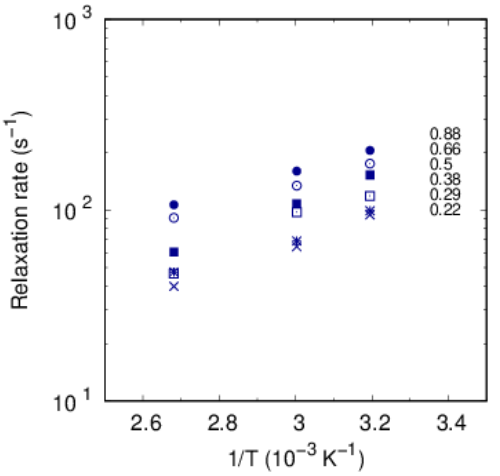

Figure S2. Arrhenius plot of spin-lattice relaxation rates of NR212.

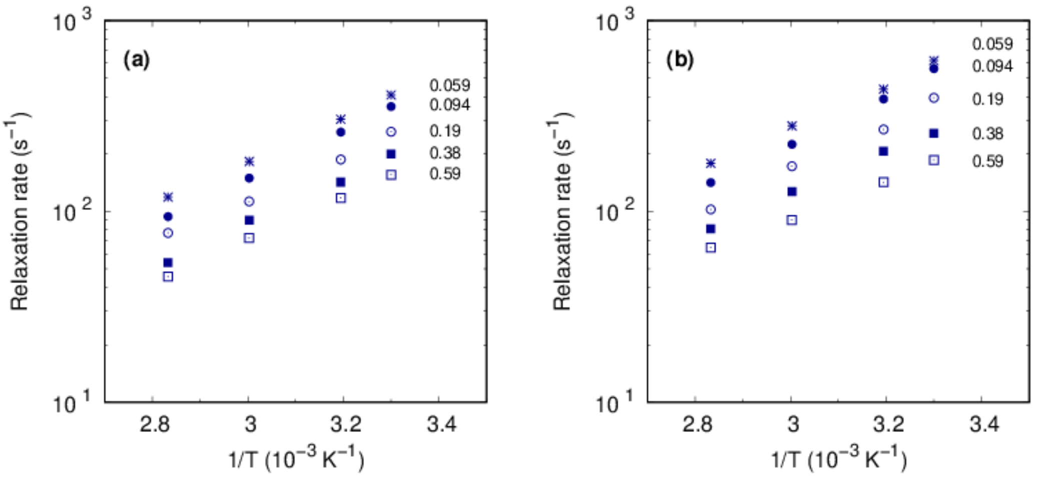

Figure S3. Arrhenius plot of spin-lattice relaxation rates of SSC-PFSA membranes.
(a) E98-05S (b) D79-12S.
